# Enhancing the online student experience through the application of Universal Design for Learning (UDL) to research methods learning and teaching

**DOI:** 10.1007/s10639-023-11948-6

**Published:** 2023-06-14

**Authors:** Mairead Seymour

**Affiliations:** Technological University Dublin, Dublin, Ireland

**Keywords:** Online learning, Asynchronous learning, Universal Design for Learning (UDL), Community of Inquiry (CoI)

## Abstract

This paper documents the process and outcomes of redesigning an online research methods module for taught postgraduate students using Universal Design for Learning (UDL). It also explores the effectiveness of UDL-informed design and practice to support the development of social, cognitive and teacher presence as defined under the Community of Inquiry (CoI) framework. The paper is based on findings from an online survey with students taking a research methods module as part of their Master of Arts (MA) programme. The findings point to a number of UDL-informed structures and practices that supported students’ engagement on the module. These include (a) accessibility of the online learning resources (b) weekly structure and signposting (c) online peer connection and collaboration and (d) lecturer communication. It was also established that UDL, as applied in the redesign of this module, supported the development of cognitive, teaching and social presence. The conclusion of this paper is that UDL-informed design and practice has the potential to influence online learning in multiple and interdependent ways; in its own right and through its application in developing cognitive, social and teaching presence. The findings highlight the benefits of adopting UDL for wider application, particularly in the context of growing diversity in student populations in higher education.

## Growing diversity in the landscape of higher education

Growing heterogeneity of the student population in higher education has brought increased focus to the diversity of students’ backgrounds, circumstances and experiences and their related learning requirements (Padden et al., 2017). In turn, it has highlighted the need for educators to respond to students’ diverse learning needs and preferences (Boothe et al., 2018; Dean et al., 2017; Lohmann et al., 2018). Universal Design for Learning (UDL) originated from the concept of universal design where the focus was on physical access to educational opportunities for learners (Fovet, 2021). Recognising that access to learning extends beyond physical access to the social, psychological, and cognitive domains resulted in the application of universal design to curriculum reform led by the Center for Applied Special Technology (CAST) in the US. Initially considered a framework to support students with disabilities or with language competency requirements, UDL has gained wider traction across the higher education sector in recent years.

As an educational framework, UDL recognises that ‘students come to learning with different experiences and will approach their learning in different ways’ (McCaughren, 2021, p.139). It represents a proactive and student-centred approach whereby learning is designed with the user in mind. The concept of equitable access is central to UDL. The idea is not that every student receives the same resources to achieve their learning goals, but rather learning is designed in ways that facilitate students’ access to the resources they require to succeed. This means designing, developing and implementing learning instruction in a way ‘that meets multiple learning needs’ (Boothe et al., 2018, p.3). UDL is therefore recognised as an enabling rather than a deficit-focused learning framework that has relevance and applicability for all students.

Based on neuroscientific research on how individuals learn, the UDL framework developed by CAST consists of three core principles providing learners with: multiple means of representation (the way information is presented to learners); multiple means of action and expression (the way learners express what they know); and multiple means of engagement (the way learners can be engaged or motivated to learn) (CAST, 2018; Meyer et al., 2014). Common to the implementation of each principle is that flexibility is embedded in the curriculum so that the diversity of learners’ needs are met. In practice, this means that learning is designed to give students options in the way they navigate information (representation), demonstrate their learning (action and expression), and connect with the learning experience (engagement). This paper focuses on the latter of these options. It details how learning was designed and implemented to sustain students’ engagement on a taught postgraduate research methods module in line with the UDL principle ‘Provide Multiple Means of Engagement’. Furthermore, it explores the effectiveness of UDL-informed design and practice in supporting social, cognitive and teacher presence as components of a positive learning experience.

## Measuring the learning experience

Garrison et al’s (2000) constructivist model of online learning - the ‘Community of Inquiry’ (CoI) model - is the lens through which students’ learning experiences are examined, and UDL-informed design is critically evaluated, in this paper. Garrison (2018, para 1) refers to the CoI model as ‘a dynamic process model of thinking and learning collaboratively’ whereby the design is not static, but adaptive and flexible to learners’ needs. This ethos resonates in the UDL model where the focus rests on designing learning in ways that meet the needs of individual learners. Furthermore, and similar to UDL, learning is an active process of inquiry within the CoI model. The CoI model provides a framework to support the design of online learning courses and e-activities, focusing on the relationships and interplay between the course instructor(s), the students, and the course content. The key premise of the model is that there are three interdependent components central to creating an effective learning experience for students: cognitive presence; social presence; and teacher presence (Saadatmand et al., 2017). These respective presences, outlined below, are utilized in this study to inform the UDL practices implemented as part of the module design process.

Cognitive presence involves student-to-student and student-to-educator interaction with content, and each other, to construct knowledge and meaning and reflect on their own learning (Garrison et al., 2000). According to Garrison et al. (2001), cognitive presence is a key aspect of supporting the development of critical thinking skills. Cognitive presence is operationalized drawing on four phases of the practical inquiry model (Swan et al., 2009). The phases include a triggering event, exploration, integration, and resolution (Garrison et al., 2000). A triggering event signals the commencement of the process and occurs when learners are exposed to a task involving a problem or challenge. In the exploration phase, learners seek out and exchange information and knowledge to address the problem or challenge and/or ‘to make sense of the experience’ (Parrish et al., 2021, p.476). Thereafter, learners move to the integration phase where they seek to tentatively assimilate new information and knowledge to develop insights, concepts and connections. According to Kilis and Yildirim (2019) this stage includes ‘convergence among community members through the connection of ideas and synthesis of information, and sustained critical reflection’ (p.180). In the final phase – the resolution phase – learners apply these insights, concepts and connections in seeking out or confirming meaningful responses to the problems or challenges raised.

Nagel and Kotzé (2010, p.46) explain that social presence is an indicator of feeling part of a community of learners – typically a class or cohort group – and being able to meaningfully contribute within that community in a way that fosters collaboration and advances learning. According to Garrison et al. (2000), social presence consists of three components: emotional expression; open communication; and group cohesion. Emotional expression refers to feeling comfortable to express feelings related to learning within a learning group. Open communication entails being responsive to others’ contributions including asking questions, expressing agreement, and continuing group discussion (Saadatmand et al., 2017) while group cohesion relates to activities that encourage and sustain collaboration. Garrison et al. (2000) suggest that group cohesion supports the development of critical thinking and expression when students see themselves as part of a group, rather than as individual learners.

Results from a survey of 30 participants on an eight-week professional development online course at three universities in Sweden found that where students perceived social presence was high, they had high levels of social interaction and connection with their peers (Saadatmand et al., 2017). Garrison et al. (2000) suggest that social presence in its own right encourages student engagement and participation, but it is also important in supporting cognitive presence by ‘indirectly facilitating the process of critical thinking’ through collaboration among learners (p.89). They argue that cognitive presence is sustained where social presence is strong. Collectively, the implication of these findings is that educators should strive to facilitate ways of promoting learner interaction with each other to support the development of social presence (Saadatmand et al., 2017).

Teaching presence requires the teacher to design, implement and facilitate learning in ways that connect their students to the content (cognitive presence) and to each other (social presence), to maintain and develop a presence within the online learning community, and to be open and responsive to online students’ queries and comments. Anderson (2008) describes teaching presence as having three aspects: design and organisation of learning and learning activities; facilitation of discourse; and direct instruction. Under design and organisation, teachers focus on designing the curriculum, selecting teaching methods, designing and/or curating learning activities, creating learning resources to support learning and deciding on time lines for the delivery of individual and group activities (Anderson et al., 2001). Anderson et al. (2001) also make reference to designing and organising learning in a way that students are aware of what is expected of them. They highlight the importance for students ‘to have a sense of the “grand design” of the course and reassurance that participating in the learning activities will lead to attainment of their learning goals’ (p.6). The second aspect of teaching presence known as ‘facilitating discourse’ involves the teacher taking an active role in facilitating students’ contributions to the learning community by encouraging their participation and acknowledging their input, prompting and maintaining discussion throughout the learning process, creating the conditions for a welcoming and safe learning environment, and assessing the efficiency and effectiveness of the process (Anderson et al., 2001; Kilis & Yildirim, 2019). Anderson et al. (2001) describe direct instruction – the third aspect of teaching presence – as an active process as it extends beyond the provision of content to directing students towards specific content to frame their knowledge development of a topic, checking and guiding their understanding through formal and informal feedback, directing them to learning resources, and responding to technical queries.

Khalid and Quick’s (2016) study of 73 university students who had completed at least one 12-week hybrid or fully online course highlights the value of teaching presence. Findings established that teaching presence was positively associated with course satisfaction and online students who had a high degree of interaction with their instructors tended to have higher degrees of satisfaction. Reporting on the findings of a survey of postgraduate Masters’ and Doctoral students undertaking a half-year research methodology course, Nagel and Kotzé (2010) found teaching presence to be the strongest presence with the design and organisation scoring the highest of all aspects of teaching presence. They attributed good course design to positive learning experiences and a high programme completion rate (87%) (p.50). Teaching presence has been described as having a ‘mediating role’ (Kilis & Yildirim, 2019, p.180) in the development of social and cognitive presence. Garrison and Cleveland-Innes (2005) also argue that teaching presence is an essential component in the transition from social presence to cognitive presence.

## Module design

The module that forms the basis of the design activity in this paper is a research methods module for taught postgraduate students. The purpose of the module is to provide students with the knowledge and skills to write a research proposal and to conduct original research for their MA thesis. Prior to the onset of the Covid-19 global pandemic, the module was delivered through weekly lectures on campus, however public health guidance restricted face-to-face university teaching and necessitated a pivot to online learning throughout 2020. The module was delivered between September and December 2020, six months after the initial stoppage of face-to-face teaching in March 2020. It meant that while the module was not initially designed for the purposes of online learning, there was time in the intervening period to plan and thereby reduce the shortcomings associated with emergency online learning (Hodges et al., 2020; Shin & Hickey, 2021). It was decided to redesign the module format so that teaching consisted of weekly synchronous online lectures supported by asynchronous learning tasks and structures. It is the asynchronous learning which is the primary focus of this paper. The process of redesigning the module was informed by the Analyze, Design, Develop, Implement and Evaluate (ADDIE) model of instructional design. ADDIE is a flexible model making it suitable for application in different instructional contexts (Peterson, 2003). The analysis stage involves identifying the nature of the situation and the gaps to be addressed; the design and development stages focus on building a response to the issues identified at the previous stage, while the implementation stage focuses on practical application. The final stage provides for evaluation and reflection on the changes made. The model is iterative and facilitates revisiting the earlier stages where required. It is the dynamic and flexible nature of the ADDIE model that has seen it utilized globally in many contexts since its development in the 1970s (Almelhi, 2021; Budoya et al., 2019; Ngussa, 2014; Patel et al., 2016). The following outlines the application of the ADDIE model in this study:

### Analysis

The research methods module was undertaken by two groups of taught postgraduate students (n = 28) located within a social sciences university department. They completed the module as part of the requirements for their MA award. The analysis stage of the re-design process considered the characteristics of the students to identify how best to support their learning. Students came from diverse disciplinary backgrounds ranging from education to humanities, social sciences, law and psychology. A number commenced their MA studies on a full-time basis following their undergraduate degree, while others had an established professional role and were returning to education on a part-time basis. Many balanced their study with part- or full-time employment and other commitments including caring responsibilities. In a few cases, English was not the students’ first language and a small number reported a specific learning disability which impacted their learning experience.

### Design

Existing research points to the challenges for students in adapting to learning in an online environment (Farrell & Brunton, 2020; Stone & O’Shea, 2019). In their study of online students, Stone and O’Shea (2019) report challenges such as feeling isolated, not having a sense of belonging or connection to the university, navigating content, and difficulties with technology. Concern about the challenges of online engagement for students consolidated the decision to use the UDL principle ‘Provide Multiple Means of Engagement’ as the foundation of the design activity in this study. This principle has been referred to as the motivation for learning or the ‘why’ of learning (Rose & Meyer, 2002). Meyer et al.’s (2014) reflection on a synthesis of research highlights the relevance of engagement to learning in concluding that ‘participation matters, involvement matters, and participation and involvement affect engagement, which in turn affects student learning’ (p.70).

The next decision was to consider how the principle ‘Provide Multiple Means of Engagement’ would be operationalized (see Table 1). A series of guidelines and checkpoints guide educators when applying UDL principles in practice (CAST, 2018). Drawing on the CoI model, specifically cognitive, teaching and social presence respectively, the two guidelines selected to inform the design activity were (1) Provide options for recruiting interest and (2) Provide options for sustaining effort and persistence (CAST, 2018).

It could be argued that the first guideline ‘Provide options for recruiting interest’ most closely aligns with cognitive presence as outlined in the CoI model (Garrison et al., 2000). Under this guideline, UDL proponents argue that where information provided to learners does not connect with them at a cognitive level, it compromises their learning as relevant information is missed and remains unprocessed. Consequently, educators must recognise that learners differ in what captures their attention and provide alternative ways to recruit their interest and engagement (CAST, 2018). Under each guideline, a number of checkpoints are outlined which provide further guidance for application in practice. The checkpoint selected under the guideline ‘Provide options for recruiting interest’ was ‘Optimize relevance, value, and authenticity’ and refers to educators recruiting interest by demonstrating the relevance of learning objectives through meaningful learning activities (CAST, 2018).

The second guideline ‘Provide options for sustaining effort and persistence’ acknowledges that learners differ in their ability to sustain ‘attention and effort’ which is an essential component for positive learning experiences and outcomes. The implication is that educators should devise alternative strategies to support students’ differing capacities to maintain their interest and learning efforts (CAST, 2018). Two checkpoints were selected under this guideline. The first checkpoint ‘Heighted salience for sustaining effort and persistence’ refers to the challenges some learners encounter in remembering their learning goals and maintaining focus on them. It points to the importance of providing regular reminders of these goals as a strategy to support students to sustain effort and focus. Examples include highlighting learning goals in different ways for learners and/or providing them with opportunities to restate them. This checkpoint closely aligns with teacher presence as articulated under the CoI model where the active role of the teacher is closely associated with positive learning experiences (Anderson et al., 2001). The second checkpoint ‘Foster collaboration and community’ is based on the premise that peer support is a valued source of support to help learners sustain interest and engagement. Educators are therefore advised to provide options for peer communication and collaboration through strategies such as creating structured learning groups or communities of learners. The implications strongly echo those outlined as part of social presence in the CoI model and reiterate the importance of peer involvement in the learning process (Garrison et al., 2000).

**Table 1:** Multiple Means of Engagement: Applying the principle in practice and alignment with Community of Inquiry (CoI) model

UDL guideline	UDL checkpoint	Activities	CoI presence
Provide options for recruiting interest (Guideline 1)	Optimize relevance, value and authenticity	Weekly preparation document & tasks Weekly email communication Group discussion Redesign of the VLE module interface	Cognitive presence
Provide options for sustaining effort and persistence (Guideline 2)	Heightened salience of goals and objectives	Weekly preparation document & tasks Weekly email communication Instructional e-recordings	Teaching presence
	Foster collaboration and community	Weekly preparation document & tasks Weekly email communication Group discussion Redesign of the VLE module interface	Social presence

### Development

The development stage involved structuring and organising the module in a way that would best embed the principle ‘Provide Multiple Means of Engagement’ and the related guidelines and checkpoints selected at the design phase. It was decided to do this by augmenting learning from the weekly online lecture sessions by developing asynchronous online weekly learning tasks and asynchronous online weekly communications to students. The rationale was that they would facilitate signposting to the relevance of key concepts and skills in advance of the online lecture sessions and support their application in practice through assigned tasks. The decision was also taken to structure asynchronous communication via email and the Virtual Learning Environment (VLE) so that students received weekly communication about what was expected of them. The purpose was to accommodate students’ differing abilities to engage and connect with the module information, and to initiate and sustain their interest and learning efforts over time (CAST, 2018). The final decision at this stage was to develop online structures that supported options for peer communication and collaboration. This was in response to the importance attached in the UDL framework to peer support as a way of sustaining engagement and enhancing learning (Kumar & Wideman, 2014).

### Implementation

The first implementation action was to redesign the entire module interface in the VLE. This redesign involved clearer delineation of topics into folders, signposting, and the addition of accompanying images to communicate content and to capture student interest. A series of audio-visual recordings were also made to support students’ access to the wider content using the screencasting and video editing software Screencast-O-Matic. Recordings on accessing relevant databases, information repositories, and previous research theses were developed and uploaded to the VLE. The overall purpose of the VLE redesign was to remove barriers to engagement by improved accessibility to the learning content.

Nagel and Kotzé (2010) argue that design should focus on instructional activities ‘that deeply engage the mind of the learner’ (p.46). With this in mind, the second implementation step was to provide students with weekly asynchronous activities to undertake in advance of live online lecture sessions. These activities were directly linked to the lecture theme which in the context of the module extended from critical reading and thinking, to research planning and design, and from methodology and methods to research ethics and analysis. The asynchronous online activities drew on a range of text-based, audio, and audio-visual learning resources e.g. a quiz, podcasts, a film, recordings with related transcripts, completing a live survey, case studies, and/or a recording with an accompanying reading. Students had choice in the online resource selected for each task, or where the resource was assigned, these were accessible in audio-visual and text format. Asynchronous activities were communicated to students through a document titled ‘Preparation for Next Week’ which was posted to the module VLE after each online lecture. This document outlined the topic for the following week, introduced students to the preparatory work, and explained why it was relevant. The rationale for the online asynchronous activities was to support and maintain student engagement throughout the 12-week module.

The third implementation step involved structured online group activities which were designed to provide a focal point for student discussion. Examples included drafting a research plan or resolving an ethical dilemma. Initially such activities were embedded throughout the live sessions whereby students would be assigned to a breakout room within the online classroom for a specified period of time. However, student feedback identified that the timed nature of breakout rooms interrupted the flow of discussion. Group collaboration was therefore moved to the end of the online lecture where students had greater control over the length of discussion time. Instead of verbal feedback, students posted key points from the discussion using the collaborative virtual noticeboard Padlet or assigned rapporteurs provided group feedback through email. Locating group discussion towards the end of the live sessions also provided more flexibility for students who so wished to engage in social conversation after the formal learning task(s) and discussion was completed.

The final implementation step underpinned the entire module and related to the lecturer’s availability to students on the module. In addition to the above mentioned activities, the lecturer emailed students a few days before each live online session with a reminder about the topic and where the topic fitted within the module and its relevance to their overall learning goals. The email also provided a reminder to students about the preparatory work to complete in advance of their weekly online session. The purpose was to maintain connection with students, to guide their learning, and to support them with remaining engaged in the learning process. Students were also encouraged to contact the lecturer where they required clarification or confirmation throughout the module.

### Evaluation

The focus of the evaluation stage was on exploring the design and implementation of learning in line with the UDL principle ‘Provide Multiple Means of Engagement’ and establishing the effectiveness, or otherwise, of UDL-informed design and practice in supporting social, cognitive and teacher presence as components of a positive learning experience.

In this study, consideration was given to utilising focus groups to facilitate open-ended discussion and produce more authentic student-led accounts of their experiences (Flick, 2018). However, the researcher was the students’ lecturer and this led to concern that students may feel obliged to take part, despite assurances that non-participation would not have adverse implications. A further concern was that students may be less willing to be critical of the lecturer’s practice where their responses were potentially identifiable. For these reasons, an anonymous online survey was selected as the data collection method to alleviate concerns about coercion and involuntary consent (see also Rao et al., 2015).

O’Leary (2017, p.227) points to ‘gathering in-depth data’ as a shortcoming of the survey method and so to reduce this challenge, the researcher elected to design a survey that incorporated closed and open-ended questions. Survey questions were structured around the quality of communication and accessibility to learning related materials and information on the VLE interface; the weekly preparation document including the weekly tasks completed off-line and weekly emails; and group discussion and collaboration with peers. Respondents also had opportunities to explain their responses and to include new perspectives through the open-ended questions in the survey.

An invitation to participate, including details of the research and a link to the survey, was emailed to students’ class email address in March 2021. Students indicated their willingness to consent through a tick-box option at the beginning of the survey. All students had completed the module and had received their final grades and feedback before being approached about the study. Just under half of the 28 students responded (46%, n = 13).

Quantitative responses were inputted into Microsoft Excel for analysis. The modest nature of the sample size meant that the analysis was focused primarily on generating descriptive statistics (Creswell, 2009). Qualitative responses were transcribed and analysed using a manual process of deductive and inductive coding (Creswell, 2009). Deductive codes were drawn from key concepts within the UDL and CoI literatures, while inductive codes were grounded in the students’ unique perspectives. Codes were merged into categories of meaning, and alongside the descriptive statistics, the data collectively informed the research question and generated new insights and implications for practice.

## Findings and discussion

Students’ perspectives on their learning experiences of UDL-informed online structures and strategies under the principle ‘Provide Multiple Means of Engagement’ are outlined below, before discussing the influence of UDL on the development of cognitive, social and teaching presence.

### Students’ perspectives on their learning experiences of UDL-informed online structures and strategies

In total, 85% of students (n = 11) stated that the weekly online asynchronous learning tasks ‘always’ or ‘often’ supported the development of their research knowledge and skills and 77% (n = 10) stated that they ‘always’ or ‘often’ enhanced their understanding of the lecture topics. With one exception, students indicated that they were able to relate the weekly tasks to their research topic, to the development of their methodology and/or to another aspect of their learning. Students’ responses suggest that for the majority, the tasks were successful in facilitating them to connect with, and apply, the learning materials to advance their knowledge.

Analysis of the data identified a number of features that underpinned students’ connection to the learning tasks. Firstly, they referred to the interesting and engaging nature of the resources provided to complete the tasks:

The interactive resources used in weekly prep were very engaging and it was clear the lecturer was doing her best to find different resources and materials to keep the content interesting (Respondent 10).

Secondly, students were positive about the choice in the weekly tasks whereby they could select among resources assigned. The words of one student captured those of others with the following description: ‘It was helpful to be given information so you could choose what was relevant for your studies’ (Respondent 4). Having choice within the content of the tasks aligns with the very essence of UDL practice where choice underpins engagement (CAST, 2018). In addition to the nature and choice of the resources provided, the analysis points to a number of UDL-informed structures and practices that were important in supporting and sustaining students’ engagement on the module. These included (a) accessibility of learning resources (b) weekly structure and signposting (c) peer connection and collaboration and (d) lecturer communication.

#### Accessibility of learning resources

In order to complete the weekly online asynchronous learning tasks, students were required to engage with assigned resources. Proponents of UDL argue that learning resources must be accessible as information not accessed is information not utilized (CAST, 2018). When asked about the instructions provided to complete learning tasks, 77% (n = 10) of students said they were ‘always’ or ‘usually’ accessible and with one exception, students indicated that the instructions were ‘always’ or ‘usually’ clear. More broadly, 85% (n = 11) of students said they could ‘always’ or ‘usually’ access the learning information required from the VLE platform without difficulty. With one exception, students positively rated the layout and ease of navigation and access on the VLE, and all were favourable about the visual appearance of the platform. These findings suggest that the removal of barriers to accessing resources helped most students to connect with the learning resources and played an important part in supporting their engagement.

#### Weekly structure and signposting

All student respondents (n = 13) indicated that the weekly preparation document uploaded to the VLE after each lecture was ‘very useful’ or ‘useful’ in signposting them to the topic for the following week and explaining the preparatory learning task(s) to be completed in advance. Students also rated the usefulness of the preparation document in helping them to stay focused on a weekly basis and highlighting the relevance of the topic in advance of the lecture session.

In addition, the follow-up weekly reminder email circulated to students by their lecturer a few days prior to their online lecture was positively rated. With one exception, students rated it as useful in providing a reminder of what would be covered at the next lecture; providing a reminder of the preparatory work to complete; and helping them to stay focused. From what students said, the structured nature of online weekly communications and related tasks facilitated them to manage their learning and remain focused on their learning goals:

They helped to keep me up-to-date on the information, rather than letting it pile up (Respondent 9).

It kept me focused and encouraged me to plan my research earlier than I might have done without the tasks. It also highlighted areas I needed to be aware of, particularly when trying to develop my research question (Respondent 11).

The nature and extent to which students perceived that the online weekly structured communications help them to sustain focus and connection with the module content demonstrates the effectiveness of this UDL-informed strategy to support their engagement. It is also important as sustaining student engagement is identified as a key facet of enabling students to achieve their learning goals under the UDL framework (CAST, 2018).

#### Peer connection and collaboration

Just two participants (15%) said that group discussion online was not important to their learning experience, while the remainder attached importance to it. Most agreed that group discussion ‘enabled connection with peers’ (77%), ‘reduced isolation and/or anxiety’ (77%), and ‘provided a source of laughter and humour’ (69%). When asked to explain what was important about group discussion for their learning on the module, students overwhelmingly referred to the social aspects including the opportunity for communication and connection:

Just being able to communicate. Being heard and it was reassuring to hear people felt the same way you did (Respondent 1).

The chance to ask the smaller questions like when things are due and how people are approaching things, small practical stuff (Respondent 5).

A chance to connect with my student colleagues which has been difficult with online learning and feel less isolated in my learning (Respondent 11).

While students mostly referred to the social aspects of group discussion, some also alluded to group discussion as a platform for knowledge construction:

The group exercises online helped, where we were given a question or poll to work out. Helped us to see different perspectives (Respondent 4).

I think talking things through, and humour, helped [me] to understand my thought processes better (Respondent 3).

Connection as a vehicle for engagement is a central tenet of UDL practice under the principle ‘Provide Multiple Means of Engagement’; it is notable therefore that group discussion was seen by students as a positive strategy to build peer communication and collaboration. The emphasis on connection as a way of supporting engagement is echoed in Farrell and Brunton’s (2020) work on online learners in higher education in Ireland. They report that a sense of belonging is a notable influence on online student engagement and highlight that students are more likely to engage and participate in learning where they perceive positive connections with their peers.

#### Lecturer communication and facilitation

Students were asked about their lecturer’s performance in communicating online in key areas including (a) learning expectations (b) signposting to relevant learning materials (c) openness to questions and (d) facilitating peer-to-peer communication. All students rated the lecturer’s performance positively in communicating learning expectations to the group with 85% (n = 11) indicating ‘excellent’ or ‘very good’ and a further two stating ‘good’:

Lecturer’s communication was the best of all modules. Everything was clearly explained and expectations set from the start (Respondent 13).

The lecturer’s examples clearly showed how the content would be applied to research (Respondent 9).

All students indicated that the lecturer’s performance in signposting them to relevant learning materials including electronic databases was ‘excellent’ or ‘very good’. Students made reference to the written guidance and the short instructional e-recordings provided as being very supportive and resources they returned to repeatedly. This echoes the findings from previous research on the application of UDL that reports students’ preference for short concise video instructions where support was needed to complete a task (Deegan, 2021). Most students (85%) (n = 11) rated the lecturer positively when asked about responsiveness to their individual questions. With regard to the lecturer’s effort to facilitate peer-to-peer communication 92% (n = 12) of students rated it as ‘excellent’ or ‘very good’. Not only was clarity of communication rated positively, but a number of students related it to their sustained engagement on the module. This latter aspect is important in light of existing literature on educators’ accessibility in supporting student engagement (Boothe et al., 2018). Stone and O’Shea (2019) found that teacher connection with online students had a positive impact on retention figures. Similarly, Lohmann et al. (2018, p.13) report that students’ interaction with their teachers ‘helped them feel more connected to the course, valued as a student, and supported in their learning’. From a UDL perspective, educators have an integral role in facilitating connection with students, between students and with the content as part of a core strategy to maintain their engagement (CAST, 2018).

### Supporting the development of Cognitive, Social and Teaching Presence through UDL

In addition to exploring the application of UDL through the lens of the principle ‘Provide Multiple Means of Engagement’, this paper sought to explore the effectiveness of UDL in supporting cognitive, social and teaching presence. In the paragraphs that follow, the influence of UDL on the development of cognitive, social and teaching presence is discussed:

#### Cognitive presence

The emphasis within the principle of Multiple Means of Engagement on designing relevant learning opportunities applicable to students’ learning echoes in the CoI model where the development of cognitive presence is associated with relevant learning activities that critically engage learners and have real-world applicability (Nagel & Kotzé, 2010). Students’ positive appraisal of the relevance and applicability of the weekly asynchronous tasks to their research topic, research methodology, and/or other aspects of their learning indicated that they had connected and engaged at a cognitive and critical level. The diversity of resources provided to support learning, coupled with the option for students to choose those most relevant to their learning interests further strengthened cognitive presence.

The structure of the weekly online asynchronous tasks also supported the development of cognitive presence on the module. Drawing on the four phases of the practical inquiry associated with cognitive presence (Garrison et al., 2000), it is argued that the weekly tasks represented ‘triggering events’ which captured students’ interest in the first instance. Thereafter, the assigned weekly resources to address the tasks, gave students the tools for exploration of the problem individually, and collectively through group discussion. Students had consistent opportunities to build their knowledge and skills incrementally through the structured weekly tasks. Arguably, this provided a framework for integrating and developing their learning, and reaching resolution on the various research methods challenges from methodology to research design and ethics in practice.

#### Social presence

Peer cooperation and collaboration is a central component of the principle Provide Multiple Means of Engagement, and UDL-informed practices to facilitate peer-to-peer connection on this module supported the development of social presence. Most students (85%, n = 11) attached importance to online group discussion as part of their learning experience, specifically the opportunities to connect with peers, reduce their sense of isolation and/or anxiety, and work collaboratively where applicable. In addition, peer communication and connection was identified by students themselves as an important facet of their engagement. Their response to what was useful about peer cooperation and collaboration echoes the components of social presence outlined by Garrison et al. (2000) including emotional expression, open communication and group cohesion. It seemed that most students were comfortable to discuss their feelings about learning within the group and were reassured by their peers’ responses (emotional expression). Furthermore, from what they said the structured group exercises facilitated opportunities to engage in discussion (open communication) and seek agreement on the challenge or scenario presented (group cohesion).

#### Teaching presence

Under the principle of Provide Multiple Means of Engagement, educators design and organise learning to sustain engagement and develop strategies and practices that promote communication and connection with students, between students, and with the learning content and these are also highlighted as components of teaching presence under the CoI model (Garrison et al., 2000). In addition to the activities outlined above, students’ strong appraisal of the lecturer’s communication of learning expectations was notable in light of its importance in achieving learning goals as part of teaching presence (Anderson et al., 2001). Students also highly rated the lecturer’s facilitation of peer-to-peer communication and openness to their questions suggesting that learning discourse was encouraged and facilitated. This is relevant in the context of facilitating discourse as a key component of teaching presence (Kilis & Yildirim, 2019). Finally, the range of instructional written and audio-visual e-resources provided to students in the form of weekly emails and instructional recordings and the positive response to them from students, provides further evidence that UDL-informed practice on the module supported the development of teaching presence.

The UDL approach adopted in the redesign of this module, namely the application of the principle Multiple Means of Engagement, supported the development of cognitive, teaching and social presence under the CoI framework. While presented as distinct entities, the CoI literature identifies that while each presence has weighted merit in its own right, it is the interdependency between the presences – connecting students and their instructors to content and to each other – that creates positive learning experiences for students (Garrison et al., 2000, 2001; Saadatmand et al., 2017). This is reflected in the wider literature on student engagement and performance in online learning. Drawing on the results of a survey of 155 students engaged in online courses, Martin and Bolliger (2018, p.218) highlight the importance of strategies to connect learners with content, with other learners, and with their instructors to create ‘engaging learning experiences’ that facilitate successful learning outcomes.

## Conclusion

Although students’ differing personal characteristics, circumstances and experiences shape their capacity to adjust, Czerkawski and Lynam (2016) point to the role of educators and instructional designers in creating the conditions to support students’ learning success. Dean et al. (2017, p.8) argue that providing learning activities for students outside of ‘class time’ extends their opportunities for learning and reinforces engagement. In this study, the asynchronous online delivery was intended to enhance students’ engagement and connection to their learning experience against the background of the global pandemic Covid-19 and the related shift to online learning. The appeal of utilizing UDL as a framework was its relevance and applicability for all students; as Rao et al. (2015, p.36) argue ‘[b]y considering and applying UD principles to their courses, instructors can … creat[e] environments that provide options, learning scaffolds and structures for students with non-apparent disabilities, while also increasing clarity and choice for all learners’. It is well-recognised in the UDL literature and through the principle ‘Provide Multiple Means of Engagement’ specifically, that there is no optimal approach to engagement, and hence multiple options for engagement are required (CAST, 2018). Students positively appraised the application of UDL in this study and key aspects included cognitive connection to learning resources; accessibility to learning guidance and resources; lecturer communication; and social connection. These positive outcomes resonate in other research pointing to successful outcomes for higher education students where UDL has been utilised (Gronneberg & Johnston, 2015; Rao et al., 2015; Scott et al., 2015).

Measuring the effectiveness of any approach, including UDL, is integral to establishing and maintaining legitimacy in pedagogical practice. It was in this critical space that the effectiveness of UDL-informed design and practice in supporting social, cognitive and teaching presence as components of a positive learning experience was explored. Here the connection between UDL-informed design and practice and the development of cognitive, social, and teaching presence was identified. The relevance of this connection rests in the well-established relationships between cognitive, social and teaching presence and positive learning experiences for students (Swan, 2006; Saadatmand et al., 2017).

Overall, the findings suggest that UDL through the principle ‘Provide Multiple Means of Engagement’ has value in its own right in recruiting and sustaining student interest and engagement through the establishment of connections between students and their learning materials, their instructors and each other. Furthermore, UDL informed design and practice has the potential to influence learning in multiple and interdependent ways through its application in developing cognitive, social and teaching presence as articulated under the CoI framework. The findings highlight the benefits of adopting UDL for wider application particularly in the context of growing diversity in student populations progressing to higher education.

## Data Availability

The datasets used and/or analysed during the current study are available from the corresponding author on reasonable request.

## Declarations

The empirical study that forms the basis of the submitted journal article was approved by the TU Dublin Research Ethics Committee. Informed consent was sought by providing student respondents with a detailed information letter about the study and the option to anonymously opt in/opt out of the study.
